# Oligopeptides/DNA Coacervate Droplets as Macromolecular Delivery Microcarriers

**DOI:** 10.1002/advs.75691

**Published:** 2026-05-13

**Authors:** Linyi Zhang, Chong Wang, Mengqi Han, Yi Yang, Teng Ma, Luoran Shang

**Affiliations:** ^1^ Shanghai Key Laboratory of Medical Epigenetics Shanghai Xuhui Central Hospital, Zhongshan‐Xuhui Hospital International Co‐laboratory of Medical Epigenetics and Metabolism (Ministry of Science and Technology Institutes of Biomedical Sciences) Fudan University Shanghai China; ^2^ Department of Rheumatology and Immunology School of Biological Science and Medical Engineering Nanjing Drum Tower Hospital Southeast University Nanjing China; ^3^ Department of Cardiovascular Surgery Ruijin Hospital Shanghai Jiao Tong University School of Medicine Shanghai China; ^4^ Department of Thoracic Surgery Zhongshan Hospital Fudan University Shanghai China

**Keywords:** chemistry, coacervate, drug delivery, intracellular drug delivery, macromolecule, phase separation process

## Abstract

Macromolecular therapeutics demonstrate significant advantages and potential for treating diverse diseases. However, their intrinsic physicochemical properties often hinder the selection of suitable carriers for efficient intracellular delivery. Here, we develop coacervates formed via liquid–liquid phase separation of oligopeptides and DNA as macromolecular carriers for cellular membrane translocation. These materials enable the efficient recruitment and release of biomacromolecules, including proteins and enzymes. Notably, our experimental data suggest that their uptake may not be entirely identical to classical endocytic pathways and instead involves cholesterol‐dependent lipid raft interactions, indicating a mechanism that may be distinct from canonical clathrin‐mediated endocytosis. Upon reaching the cytoplasm, the DNA component of the coacervates is degraded by intracellular DNA‐processing enzymes, leading to coacervate disassembly and subsequent release of the therapeutic macromolecules. Together, these coacervates establish a generalizable platform for intracellular delivery of macromolecular therapeutics, integrating membrane translocation with programmable cytosolic release.

## Graphical abstract

Oligopeptide–DNA coacervates formed via liquid–liquid phase separation serve as programmable carriers for macromolecular cargos, entering cells through lipid raft–associated pathways and subsequently undergoing enzyme triggered intracellular disassembly driven by DNA degradation processes, thereby enabling efficient, controlled, and spatially defined release of biomacromolecules within the cytoplasmic environment for intracellular delivery applications.

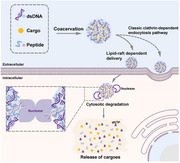

## 1 Introduction

Macromolecular therapeutics, including nucleic acids, proteins, and peptides, have emerged as powerful modalities for the treatment of cancer, cardiovascular and metabolic diseases, autoimmune disorders, and genetic abnormalities [1, 2, 3]. However, their clinical application is often hindered by delivery challenges due to their hydrophilic nature and large molecular size, which limit plasma membrane barrier permeability and in vivo bioavailability [4, 5]. Moreover, failure of endosomal escape for cargoes taken up via endocytosis will substantially diminish their intracellular effective concentration [6, 7, 8]. Therefore, efficient intracellular delivery strategies are essential for enhancing the therapeutic efficacy of macromolecular agents. Existing intracellular delivery strategies can generally be categorized into physical penetration and nanocarrier‐mediated penetration (including liposomes, silica nanoparticles, engineered exosomes, etc.) [9, 10, 11]. Despite the extensive research efforts dedicated to these delivery strategies, they have certain limitations. For instance, mechanical approaches, such as fluid shear force application, can transiently increase membrane permeability by disrupting the cell membrane structure [12, 13, 14]. However, these methods carry a risk of irreversible membrane damage, which may adversely affect cell viability and functionality. For nanocarrier‐mediated penetration, the synthesis and preparation of certain nanocarriers involve intricate procedures, and some nanocarriers may induce cytotoxic responses, thereby presenting challenges in their translation to in vivo applications and clinical settings [15]. Collectively, these limitations underscore the importance of developing intracellular delivery approaches that are both noninvasive and practically accessible in terms of preparation and application.

Coacervates, which arise from liquid–liquid phase separation (LLPS), have gained prominence as versatile and promising platforms for drug delivery due to their ease of preparation and enhanced biocompatibility afforded by their all‐aqueous environment [16, 17, 18, 19, 20]. Fundamentally, these materials can be classified into two primary categories: complex coacervates, which assemble via the interactions of two or more components; and simple coacervates, which are driven by the self‐interaction of a single component [21, 22, 23, 24]. Regardless of their structural composition, coacervates feature a uniquely all‐aqueous environment that enables facile preparation, superior biocompatibility [25, 26, 27], and selective partitioning of biomacromolecules while precisely preserving their intrinsic bioactivity [28, 29, 30]. Consequently, coacervates are increasingly investigated as advanced vehicles to deliver macromolecular therapeutics [31, 32, 33]. Importantly, previous studies have reported that coacervates can be internalized via a lipid raft–mediated pathway, which helps bypass conventional endosomal entrapment and escape, thereby enabling efficient cytoplasmic delivery [16, 34]. Once inside the cell, the intrinsic programmability of coacervates can be harnessed [35]. By designing building blocks that respond to intracellular cues such as ubiquitous enzymes or redox agents, targeted degradation can be triggered, enabling precise spatiotemporal release of encapsulated payloads and thereby maximizing therapeutic efficacy [17, 36].

**FIGURE 1:**
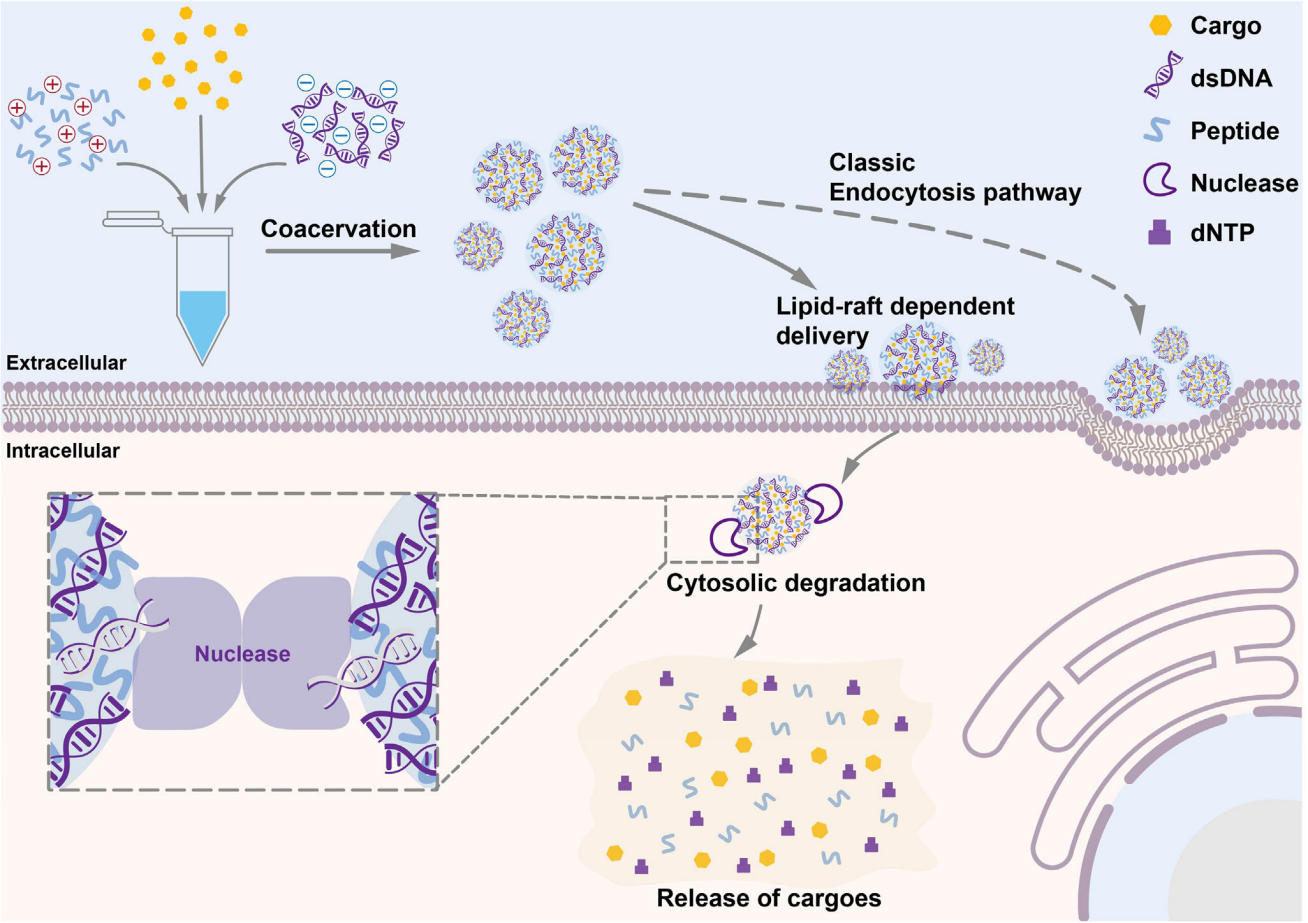
Oligopeptides/DNA coacervate droplets for intracellular macromolecular drug delivery. Oligopeptides spontaneously undergo LLPS with DNA, forming dynamic coacervate droplets capable of efficiently encapsulating biomolecular cargos. Upon interacting with living cells, these droplets could be internalized via a lipid raft‐dependent pathway. Once inside the cytoplasm, cellular nucleases recognize and degrade the DNA scaffold of coacervate droplets, enabling precise and timely cargo release.

Herein, building upon these principles, we propose a rationally designed coacervate‐based microcarrier for the intracellular delivery of macromolecules, as illustrated in Figure 1. Given the presence of cellular enzymes specialized in degrading exogenous DNA [37, 38], we devised a coacervate‐based delivery system using DNA as a scaffold molecule. To promote coacervation with negatively charged DNA, we selected and evaluated a diverse range of cationic oligopeptides (K10, R10, RA1, and RA2) as complementary scaffold molecules [39]. Through comprehensive material characterization and molecular dynamic simulations, we conclusively determined that the coacervates formed by R10 and DNA possess the most promising potential as intracellular macromolecular carriers. Systematic pharmacological inhibition studies demonstrated that their uptake differs in part from classical clathrin‐mediated endocytosis and instead involves cholesterol‐dependent lipid raft interactions. After cellular internalization, these DNA‐based coacervates could be recognized and degraded by intracellular nucleases, which leads to the disassembly of coacervate droplets and subsequent release of the encapsulated cargos. Intracellular delivery of functional proteins, including fluorescent reporters and functional enzymes, is achieved using this platform. These findings provide a novel and versatile carrier system for biomacromolecule delivery, with a rational design framework leveraging the intrinsic enzymatic machinery in cells and providing mechanistic insights into coacervate‐cell interactions.

## 2 Results

### 2.1 Formation and Characterization of Oligopeptides/DNA‐based Coacervate Droplets

**FIGURE 2:**
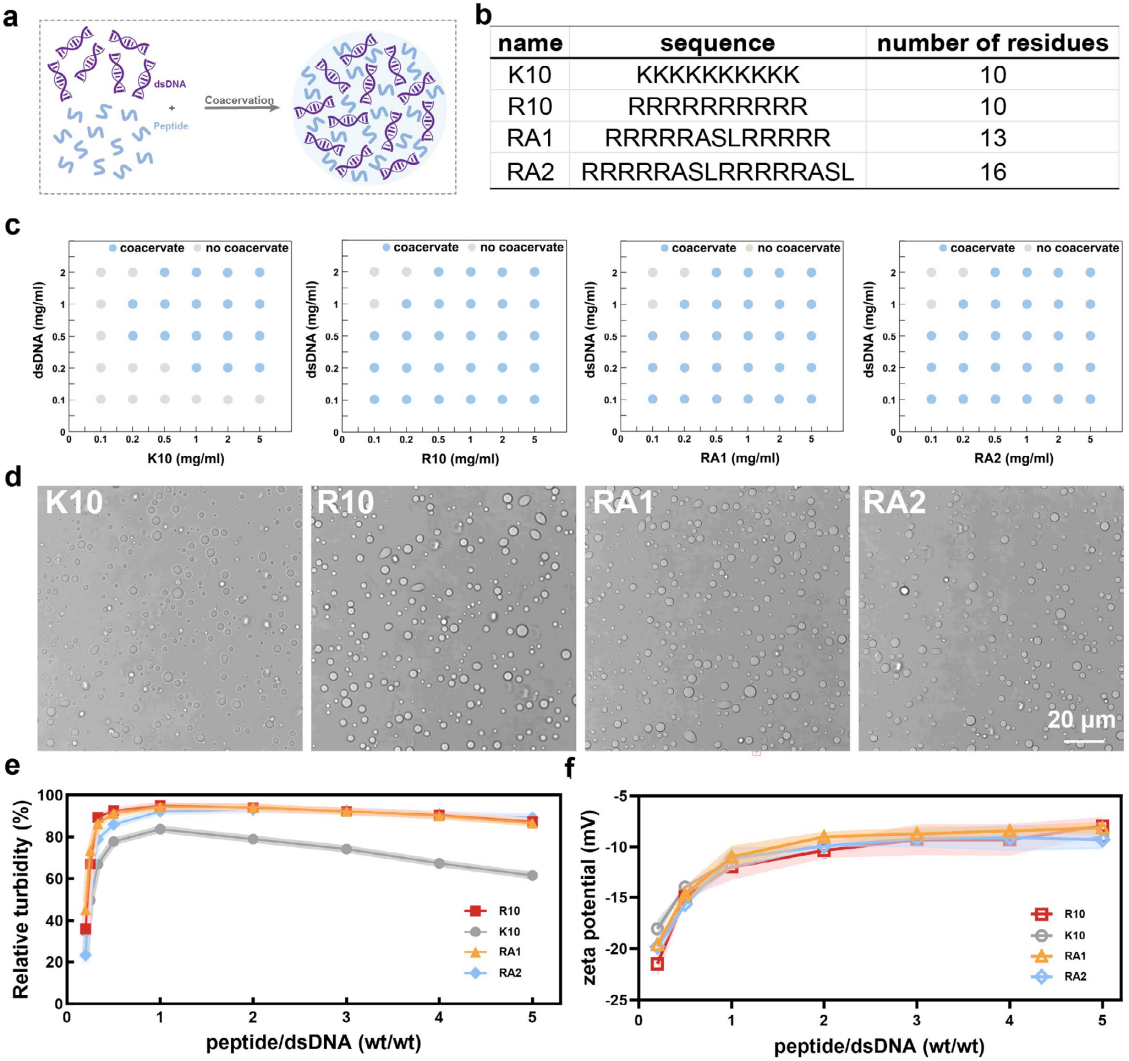
Formation and characterization of oligopeptides/DNA coacervate droplets. (a) Schematic illustration of coacervate formation by peptide and DNA. (b) Designation, amino acid sequence, and residue number of the peptides used. (c) State diagrams of coacervates formed by K10, R10, RA1, and RA2 in the presence of DNA. Blue dots denote successful phase separation, whereas grey dots indicate no observable phase separation. The concentrations shown in the diagram represent the respective concentrations of the two components before they are mixed in equal volumes. (d) Representative optical microscopy images of coacervate droplets formed by DNA and the corresponding peptides. The concentration of DNA and peptides are 10 mg/mL and 1.4 mg/mL, respectively, with the mass ratio of DNA and peptides being 1:1. (e) Relative turbidity of coacervates formed by K10, R10, RA1, or RA2 with DNA at various mass ratios. Data represent mean ± s.d. (*n* = 3). (f) Zeta potential measurements for coacervates formed by K10, R10, RA1, or RA2 with DNA at various mass ratios. Data represent mean ± s.d. (*n* = 3).

To construct a controllable complex coacervate system (Figure 2a), we selected genomic DNA extracted from salmon sperm as the anionic scaffold molecule. For the cationic polyelectrolyte component, a series of oligopeptides composed primarily of basic amino acids, including K10, R10, R5ASLR5 (RA1), and R5ASLR5ASL (RA2), were systematically screened and comparatively analyzed (Figure 2b). First, we investigated the ability of these four oligopeptides to form complex coacervates with DNA in a phosphate‐buffered saline (PBS) environment and drew state diagrams to characterize the formation of complex coacervate droplets at varying component concentrations and mixing ratios (Figure 2c). The images of the corresponding coacervation droplets were shown in Figure 2d. Among the four tested oligopeptides, K10 exhibited the narrowest concentration window for effective phase separation, with droplet formation observed only at concentrations over 0.2 mg/mL.

In contrast, the other three oligopeptides (R10, RA1, and RA2) were capable of forming coacervate droplets over a significantly broader concentration range (0.1–5 mg/mL), which may be attributed to their stronger interactions with DNA, thereby enhancing phase separation capability. Due to the fact that the pKa value of lysine (10.5) is lower than that of arginine (12.5), and molecular structures of the two peptides are different, under physiological pH conditions (7.2–7.4), the guanidino group on the side chain of arginine is more likely to undergo complete and stable protonation than the ε‐amino group on the side chain of lysine. Therefore, arginine has a higher positive charge intensity, making it more prone to engage in strong electrostatic interactions with negatively charged moieties, such as the phosphate groups of nucleic acids.

Furthermore, we prepared a series of solutions with varying content of peptide to DNA and measured their turbidity. The solution volume was kept at 200 µL; 2 mg/mL peptide and 2 mg/mL DNA solutions were mixed at mass ratios ranging from 0.2 to 5 (peptide to DNA) (Figure 2e). The four oligopeptides reached maximum turbidity at mass ratios of 1 (R10, K10, and RA1) or 2 (RA2). Moreover, except for K10, the other three oligopeptides maintained a relatively high turbidity at high mass ratios. Zeta potential measurements indicated that the droplet suspensions formed by DNA and these four oligopeptides were negatively charged across all mass ratios tested (Figure 2f). Notably, as the relative concentration of the positively charged components within the coacervate increased, the Zeta potential became less negative.

### 2.2 Characterization of Dynamic Properties of Coacervate Droplets

**FIGURE 3:**
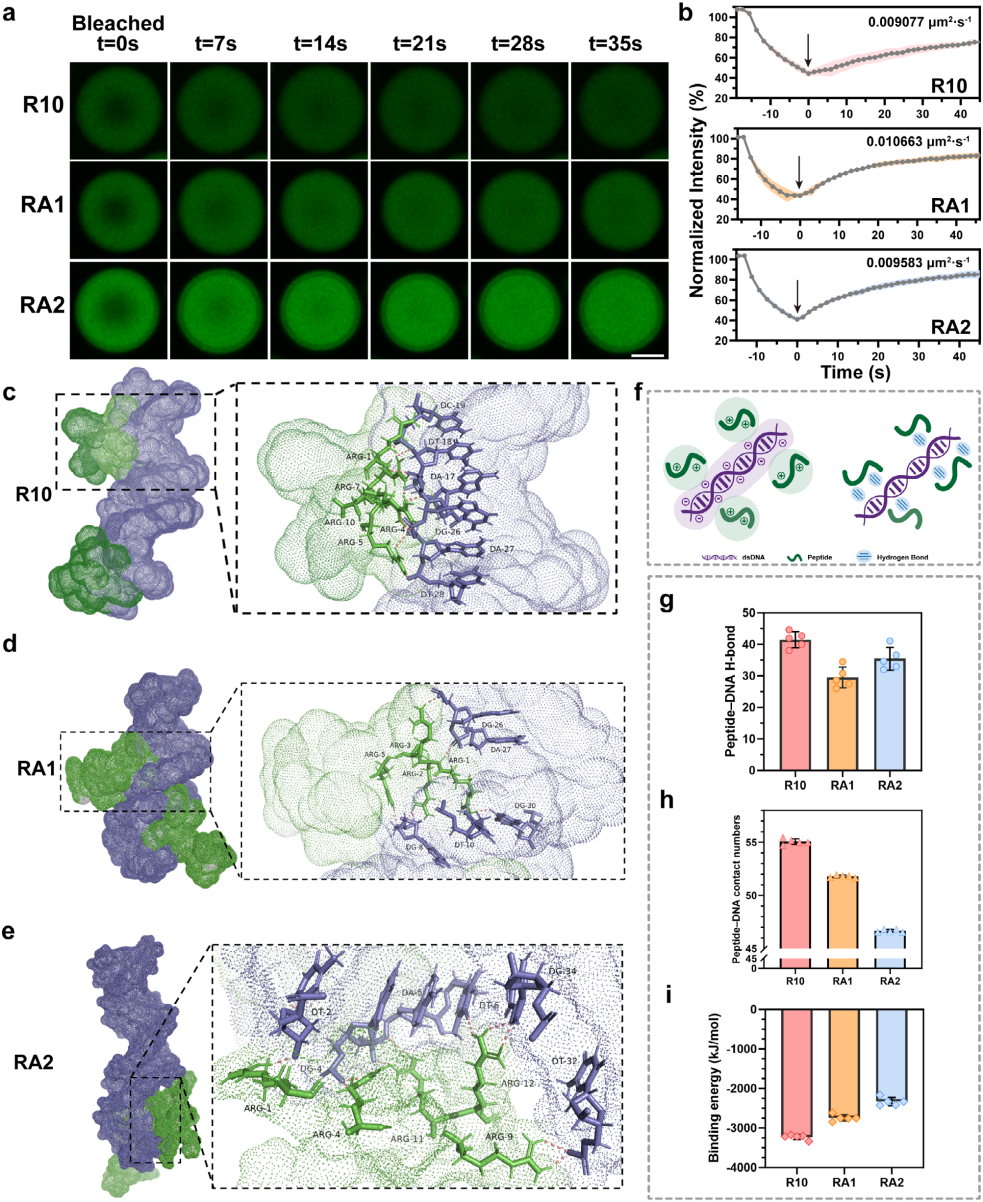
Dynamic properties and molecular interaction mechanisms of peptide/DNA coacervate. (a), Confocal microscopy images of coacervate droplets during the FRAP experiment. scale bar, 2 µm. (b), Quantitative analysis of fluorescence intensity at the photobleached region of coacervate droplets during FRAP. Arrows denote the time point of photobleaching completion and the initiation of fluorescence recovery. Data are displayed as mean ± s.d. (*n* = 3). (c–e), Representative snapshots showing equilibrium conformations of R10/DNA (c), RA1/DNA (d), and RA2/DNA (e) coacervates obtained from AA‐MD simulations. DNA is shown in purple, oligopeptides in green, and hydrogen bonds are represented by pink dashed lines. (f) Schematic illustration of the primary intermolecular interactions within peptide/DNA coacervates. (g–i) Quantitative energy analysis of hydrogen bonding (g), peptide‐DNA contact numbers (h), and binding energy (i) of various peptide/DNA coacervates by AA‐MD stimulation. Data represent mean ± s.d. (*n* = 5).

Based on preliminary screening experiments, R10 and its derivatives (RA1 and RA2) formed highly concentrated and stable coacervate droplets with DNA under broader physicochemical conditions than K10. We next investigated the dynamic properties of these three peptide/DNA coacervate systems by fluorescence recovery after photobleaching (FRAP) using fluorescein isothiocyanate (FITC)‐labeled peptides. FRAP time‐lapse imaging revealed time‐dependent recovery of fluorescence intensity in the bleached region (Figure 3a), indicating sustained molecular exchange capability and liquid‐like property within the coacervates. Quantitative analysis of the fluorescence recovery curves (Figure 3b) showed that R10/DNA coacervates display slower recovery kinetics compared with RA1/DNA and RA2/DNA systems, indicative of reduced internal fluidity. In agreement with this, the diffusion coefficient of R10/DNA (0.009077 µm^2^ /s) is lower than that of RA1/DNA (0.010663 µm^2^/s) and RA2/DNA (0.009583 µm^2^/s), suggesting stronger intermolecular interactions between R10 and DNA.

To elucidate the molecular basis of the differential fluidity among the coacervates formed by DNA and the three oligopeptides, all‐atom molecular dynamics (AA‐MD) simulations were performed on equilibrated peptide/DNA systems within 20 × 20 × 20 nm^3^ simulation boxes. The stick models of the equilibrium conformations (Figures 3c–e) illustrate the molecular configurations of the key interaction regions between the oligopeptides and DNA. Notably, the side chains of the arginine residues in the oligopeptides formed stable hydrogen‐bonding networks with the DNA phosphate backbone (Figure 3f). Analysis of the MD simulations (Figures 3g–i) shows that R10 forms a larger number of hydrogen bonds with DNA and exhibits higher peptide–DNA contact numbers compared with R5ASLR5 and R5ASLR5ASL. Quantitative energy analysis demonstrates that the R10/DNA system exhibited the highest binding energy (ΔG = −3228 ± 64 kJ/mol), followed by RA1/DNA (ΔG = −2744 ± 82 kJ/mol) and RA2/DNA (ΔG = −2332 ± 104 kJ/mol) systems. This trend, consistent with the FRAP and turbidity analyses, indicates stronger intermolecular interactions between R10 and DNA.

### 2.3 Assessment of Coacervate Delivery Efficiency and Mechanistic Investigation of Internalization

**FIGURE 4:**
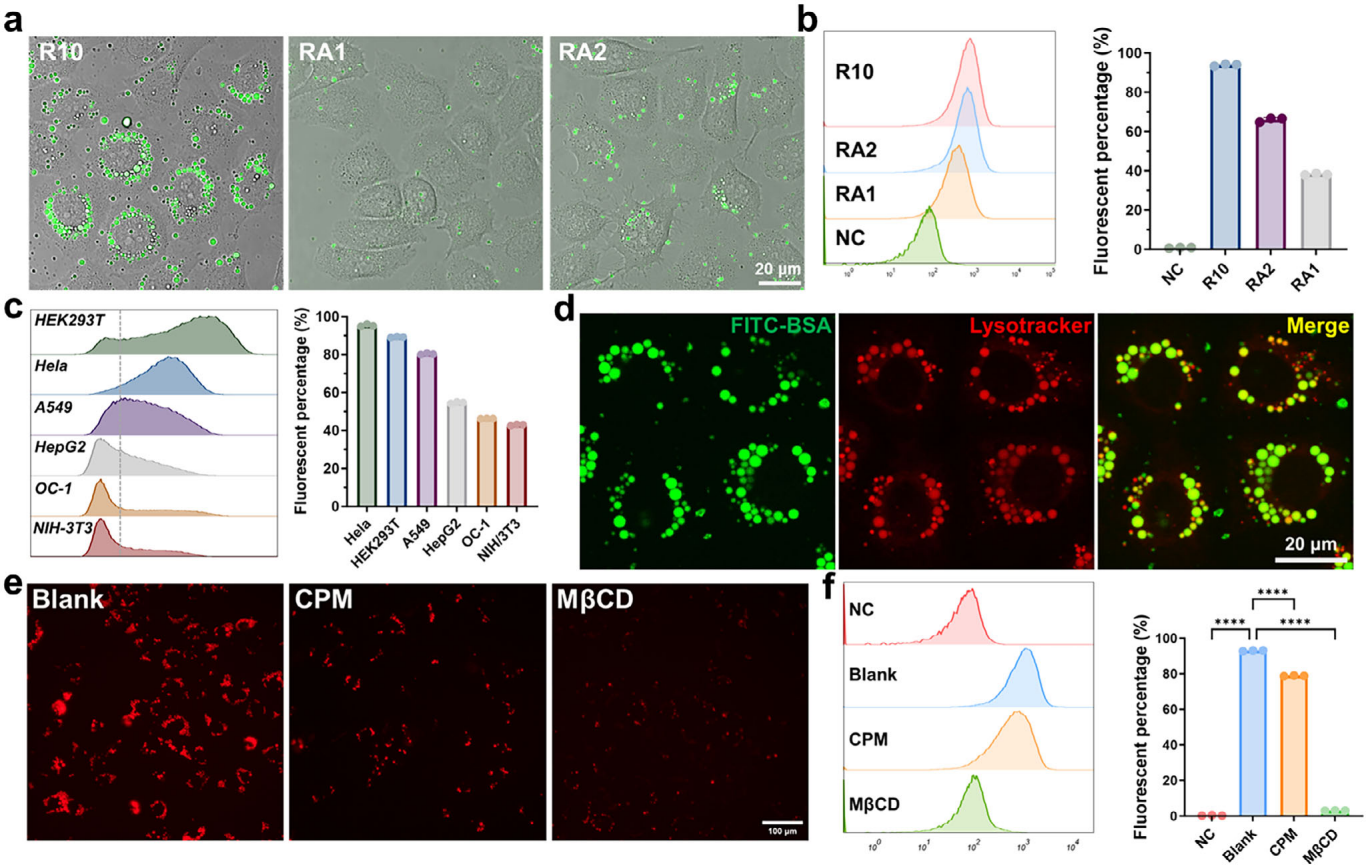
Cellular uptake and internalization of protein‐loaded peptide/DNA coacervate droplets. (a) Fluorescence microscopic images of HeLa cells treated with FITC‐BSA‐loaded R10/DNA, RA1/DNA, and RA2/DNA coacervates following a 4‐h co‐incubation, respectively. (b) Flow cytometry quantification of cellular uptake for three types of FITC‐BSA‐loaded coacervates. Values depict the mean ± s.d. (*n* = 3). (c) FITC‐BSA‐loaded R10/DNA coacervate uptake in various cell types quantified by FACS. All data points represent the mean ± s.d. (*n* = 3). (d) Confocal fluorescent images of HeLa cells following 4 h of treatment with FITC‐BSA‐loaded R10/DNA coacervate droplets. Lysosomes were stained with LysoTracker. (e) Fluorescence microscopy images of HeLa cells pretreated with pathway‐selective inhibitors (CPM or MβCD), followed by a 4‐h incubation with CY3‐loaded R10/DNA coacervates. (f) FACS analysis showing the delivery efficiency of FITC‐BSA‐loaded R10/DNA coacervates into HeLa cells pretreated with CPM or MβCD. Data represent mean ± s.d. (*n* = 3). Statistical comparisons were made using one‐way ANOVA, ^****^ *p* < 0.0001.

In previous experiments, we demonstrated that R10, RA1, and RA2 form coacervates with DNA, primarily through electrostatic interactions and hydrogen bonding. Building on these findings, we explored the potential of peptide/DNA coacervates for drug delivery by systematically investigating their cellular uptake efficiency. We incubated HeLa cells with the coacervates loaded with FITC‐BSA as a model cargo. After co‐incubation with the three types of FITC‐BSA‐loaded coacervates at equal concentrations, confocal microscopy images revealed that the R10/DNA system exhibited the highest intracellular accumulation of green fluorescent droplets within a standardized field of view, followed by the RA2/DNA system, whereas the RA1/DNA system showed minimal fluorescence (Figure 4a). To quantitatively present the differences in delivery efficiency among the three systems, analysis of fluorescence intensity was conducted by FACS (Figure 4b). R10/DNA system achieved the highest delivery efficacy (92.3%), significantly surpassing the RA2/DNA (68.5%) and RA1/DNA (45.2%) systems, which were consistent with the confocal microscopy observations. Based on these findings, the R10/DNA system was identified as the most promising candidate for efficient biomacromolecule delivery. Therefore, subsequent experiments focused on using R10/DNA coacervate droplets to further investigate their drug delivery potential. To address the concern that the coacervates may disassemble before cellular uptake, we monitored the morphology of R10/DNA coacervates over time under the same serum‐free Opti‐MEM incubation conditions used for cell exposure. Meanwhile, to assess whether the coacervates would undergo aggregation, fusion, or coalescence under crowded cytoplasm‐like conditions, we performed an in vitro crowding assay using PEG as a synthetic crowding agent [40]. Confocal microscopy showed that droplets remained detectable throughout the observation period with and without 5% PEG (Figure S1). Moreover, the average droplet area gradually increased over time (Figure S2), suggesting a certain degree of droplet coalescence.

To further evaluate the applicability of the R10/DNA coacervate system, we expanded the spectrum of target cell lines to include several cell lines that are difficult to transfect using traditional methods. Following a 4‐h transfection with the FITC‐BSA‐loaded R10/DNA coacervates, flow cytometry was employed to measure the cellular fluorescence intensity (Figure 4c). The transfection efficiency reached approximately 90% in Hek‐293T, HeLa, A549 cell lines, while maintaining ∼50% efficiency in OC‐1, HepG2, and NIH‐3T3, indicating the broad‐spectrum transfection capability of the R10/DNA system.

Previous studies have suggested that coacervate internalization may involve uptake features that are not fully captured by canonical endocytic models, including lipid raft‐dependent processes [16, 17, 41]. To elucidate the internalization mechanism of the R10/DNA coacervates, lysosomal colocalization was assessed using organelle‐specific dyes [16]. As can be observed from the confocal images in Figure 4d and Figure S3, lysosomes do not fully colocalize with coacervate droplets, although partial overlap is observed. This phenomenon may be attributed to the adsorption and enrichment of the dye by the coacervates (Figure S4). This special uptake mode may depend on the lipid raft regions and cholesterol content of the cell membrane [16, 41]. Based on this speculation, cells were treated with clathrin‐mediated endocytosis inhibitor (chlorpromazine, CPM) or lipid raft disruptor (methyl‐β‐cyclodextrin, MβCD), followed by transfection with CY3‐loaded coacervates. Representative fluorescence images (Figure 4e) suggested reduced cellular uptake after inhibitor treatment, with a visually pronounced effect in both CPM and MβCD groups. However, FACS quantification (Figure 4f) showed that CPM produced moderate inhibition, whereas MβCD caused a much stronger reduction in uptake. To further explore the integrity of coacervates under endosome‑like conditions, we examined whether acidic endosomal‐like conditions would immediately disrupt the R10/DNA coacervates. Preformed coacervates were exposed to buffers spanning pH 4–9, a range covering conditions relevant to early endosomes, late endosomes, and lysosomes [42, 43], and the relative turbidity was measured immediately after treatment (Figure S5). Notably, the coacervates retained high turbidity across all tested pH values, with relative turbidity remaining above 80%, indicating that reduced pH alone does not induce rapid bulk collapse of the R10/DNA coacervate droplets at initial exposure. Collectively, these findings suggest that R10/DNA coacervate uptake involves a lipid raft‐ and cholesterol‐sensitive pathway and is not entirely consistent with canonical clathrin‐mediated endocytosis.

### 2.4 Delivery and Release of Biomacromolecules

**FIGURE 5:**
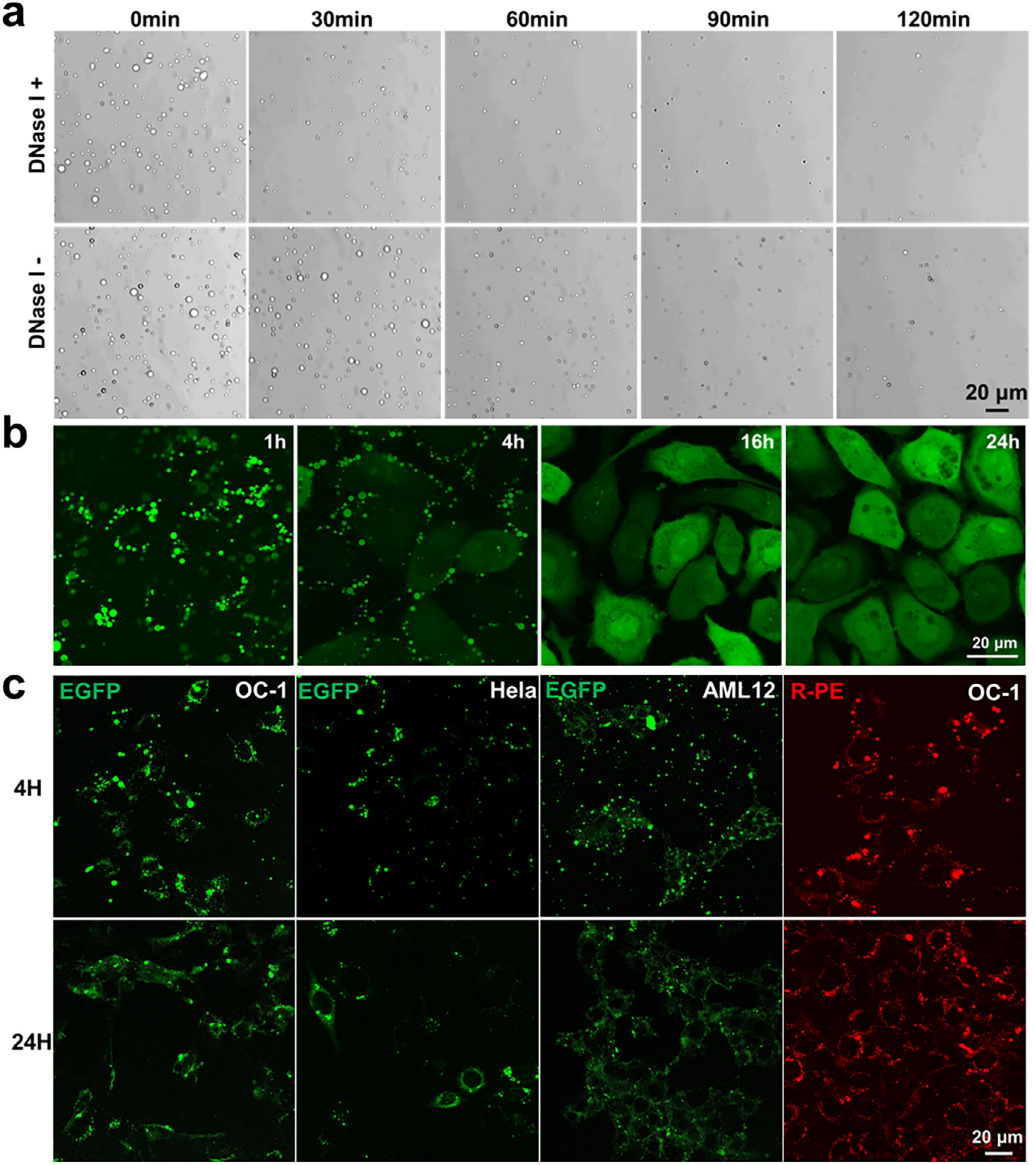
Degradation of coacervates and intracellular delivery of proteins. (a) Optical microscopy images of DNase I‐treated R10/DNA coacervate droplets and the control group at various time points within 2 h. (b) Time‐lapse confocal fluorescence imaging of HeLa cells at designated time points (1, 4, 16, and 28 h) during co‐incubation with FITC‐R10/DNA coacervate droplets. (c) Confocal fluorescence images showing the delivery of EGFP and R‐PE into OC‐1, HeLa, and AML‐12 cells after 4 and 24 h of incubation with R10/DNA coacervates loaded with EGFP or R‐PE. Representative images from three independent experiments are shown, and similar trends were observed in all replicates.

Cells possess sophisticated and tightly regulated systems for sensing and processing exogenous DNA. Central to this system are the cGAS/STING signaling pathway and exonuclease TREX1, which detect and degrade exogenous DNA within the cytoplasm. This process is vital to safeguard genomic integrity and maintain intracellular homeostasis. Based on the cytoplasmic DNA processing network, we propose that the anionic scaffold DNA within the coacervate complex can be recognized and cleared by the innate pathways of the cell. By harnessing this natural mechanism, the intracellular decomposition of coacervates can be effectively triggered, enabling controlled drug release. To test this, we systematically investigated the capacity of R10/DNA coacervates for controlled drug release by examining their degradation behavior using an in vitro experimental system. Specifically, salmon sperm DNA, which serves as a structural scaffold during coacervate formation, was treated with nonspecific DNA nucleases (DNase I). The DNA components of the coacervates could be effectively degraded by DNase I (Figure S6). Further, we investigated the degradation of coacervate droplets by treating them with DNase I for 2 h. The results showed that while coacervates underwent partial spontaneous degradation in the control group (i.e., without enzyme treatment), enzymatic degradation was more effective (Figure 5a and Figure S7). In addition, the coacervates constructed with different component ratios exhibited similar degradation characteristics (Figure S8). Based on these results, we speculate that the coacervate system may follow a similar enzymatic metabolic mechanism in intracellular environments.

To dynamically monitor the metabolic fate of coacervates in living cells, coacervates consisting of DNA and FITC‐labeled R10 were transfected into cells and tracked via time‐lapse imaging (Figure 5b). Confocal microscopy revealed distinct temporal patterns: coacervates were predominantly localized to the pericellular membrane without internalization at 1 h postincubation; sporadic green fluorescent droplets emerged with weak whole‐cell fluorescence at 4 h; after 16–28 h, the fluorescent droplets were markedly diminished, accompanied by uniformly distributed green fluorescence across most cells, indicating intracellular coacervate degradation. Control experiments using free FITC‐R10 solution showed negligible fluorescence within the cells, indicating minimal cellular uptake (Figure S9). An additional co‐culture experiment using dual‐labeled, CY3‐loaded FITC‐R10/DNA coacervates showed red fluorescence throughout all cells after 24 h (Figure S10), which colocalized with the FITC signal. In contrast, the free CY3 solution control group exhibited no such fluorescence (Figure S11), further confirming the efficient payload release following intracellular coacervate degradation.

To evaluate the biomacromolecule delivery capacity of the R10/DNA coacervate system, fluorescent proteins, EGFP and R‐PE, were selected as model macromolecules. Marked transfection efficiency was observed in HeLa, OC‐1, and AML‐12 cell lines (Figure 5c). Notably, the transfected intracellular coacervate droplets began to disassemble in some cells within 24 h, accompanied by expanded dispersion of fluorescence signals in the cellular interior, although this transition was not uniformly evident in every cell.

**FIGURE 6:**
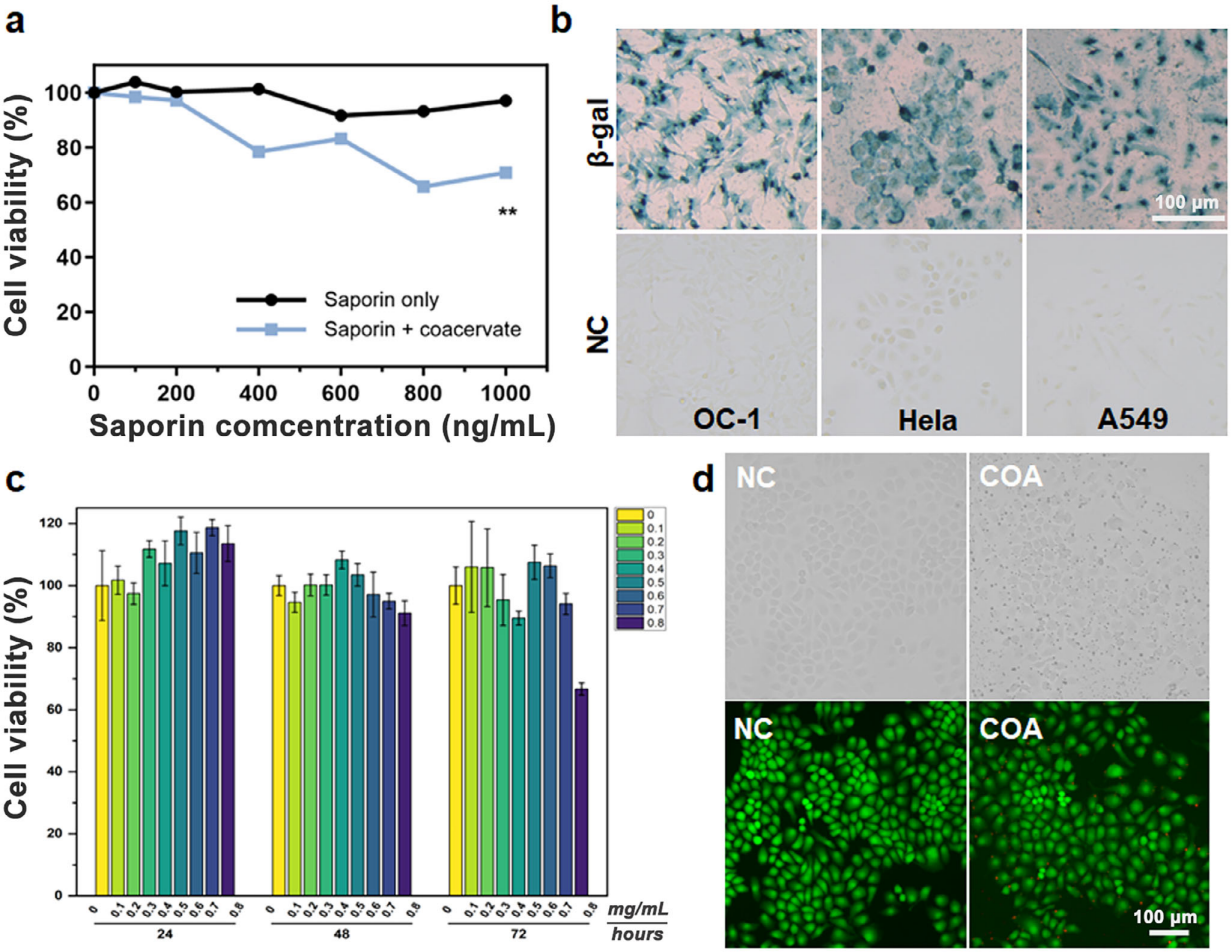
Cytocompatibility and biocompatibility analysis of peptide/DNA coacervates. (a) Cytotoxicity of free saporin and saporin‐loaded R10/DNA coacervates at various saporin doses. Results are presented as mean ± s.d. (*n* = 3). Statistical significance was assessed by a two‐sided Student's *t*‐test (^**^ *p* < 0.01). (b) X‐Gal staining of HeLa, OC‐1, and A549 cells after incubation with free β‐galactosidase (NC) and β‐gal‐loaded R10/DNA coacervate droplets. (c) Cell viability of OC‐1 cells treated with graded coacervate concentrations for 24–72 h, measured by CCK‐8 assays. Data represent mean ± s.d. (*n* = 3). (d) Calcein‐AM/PI double‐stained HeLa cells under bright‐field and fluorescence microscopy: untreated control (NC) and cells treated with 0.4 mg/mL R10/DNA coacervates (COA) for 6 h.

To further investigate whether R10/DNA coacervates could achieve intracellular delivery while preserving protein activity and function, we evaluated their delivery efficiency using a cytotoxic substance, saporin, which is a type I ribosome‐inactivating protein (RIP) derived from *Saponaria officinalis*. Due to the lack of transmembrane domains, free saporin alone showed minimal cytotoxicity, as confirmed in HeLa cells (Figure 6a). Notably, saporin delivered via R10/DNA coacervates induced a dose‐dependent reduction in cell vitality, indicating effective cellular internalization and preservation of enzymatic activity (Figure 6a). Building on these results, we next valuated β‐galactosidase (β‐gal), a large tetrameric enzyme (∼464 kDa) widely used as a reporter in gene expression and senescence assays. β‐gal catalyzes the hydrolysis of X‐Gal (5‐bromo‐4‐chloro‐3‐indolyl‐β‐D‐galactopyranoside), producing insoluble blue indigo precipitates. Cells exposed to free β‐gal exhibited no detectable X‐Gal staining, whereas cells incubated with β‐gal‐loaded R10/DNA coacervates displayed prominent intracellular blue precipitates across HeLa, OC‐1, and A549 cell lines (Figure 6b). This further confirmed the ability of the system to deliver large biomolecules intracellularly while maintaining their activity.

The biocompatibility of R10/DNA coacervates was assessed using the CCK‐8 assays combined with calcein‐AM/PI assay. CCK‐8 assay revealed that OC‐1 cells incubated with R10/DNA coacervates at concentrations up to 0.6 mg/mL for 72 h retained viability comparable to the PBS control group (Figure 6c), highlighting the excellent biocompatibility of the system. Calcein‐AM/PI double‐staining revealed intense green fluorescence with minimal red signals in cells treated with 0.4 mg/mL of R10/DNA coacervates (Figure 6d), indicating high cell viability. Collectively, these findings demonstrate that R10/DNA coacervates enable efficient intracellular delivery of functional biomacromolecules without compromising their bioactivity, positioning this platform as a promising strategy for macromolecule‐based therapeutics.

## 3 Discussion

In this study, we show that DNA undergoes liquid‐liquid phase separation with cationic peptides (RA1, RA2, and R10) to form stable coacervate droplets across a broad range of mass ratios. These coacervates efficiently encapsulate biomacromolecules, including peptides and proteins, via affinity partitioning, while the dense microenvironment preserves their bioactivity. Among them, R10/DNA coacervates exhibit high internalization efficiency across multiple cell lines, including those resistant to conventional transfection methods. Mechanistically, the cellular uptake of the R10/DNA coacervates appears to involve a lipid raft/cholesterol‐sensitive mechanism and may not be fully explained by traditional clathrin‐dependent endocytosis alone. Moreover, the partial colocalization with lysosomal compartments suggests that conventional endocytic trafficking and endosomal maturation are still likely involved to some extent. Therefore, rather than indicating a completely unique uptake route, the current data support the possibility that R10/DNA coacervates enter cells through a noncanonical, yet not fully distinct entry route. Following internalization, the coacervates gradually dissociate within the intracellular environment, enabling controlled release of the encapsulated cargos. Together, this hybrid uptake mechanism, coupled with intracellularly triggered disassembly, provides a strategy for spatiotemporally controlled delivery of biomacromolecules.

Despite these advances, several limitations remain to be addressed. First, there are technical bottlenecks in the co‐delivery of nucleic acid molecules in this system. Since DNA is one of the components of the coacervates in this system, the introduction of foreign nucleic acids may cause a shift in the critical concentration for phase separation and compromise the stability of the droplets, which necessitates subsequent optimization of the coacervate components. Second, the molecular mechanisms underlying transmembrane transport remain incompletely understood, particularly regarding the quantitative contributions of lipid raft‐mediated uptake, conventional endocytosis, and subsequent intracellular trafficking. In addition, the post‐internalization fate of the coacervates, including their intracellular processing and degradation pathways, remains to be elucidated. Third, an important consideration for peptide/DNA coacervate‐based delivery is the influence of the extracellular medium on condensate integrity. In particular, serum proteins, salts, and other medium components may alter intermolecular interactions within the coacervates and thereby promote partial or complete disassembly before cellular uptake. Future studies should focus on improving condensate robustness under serum‐containing conditions, for example, by tuning peptide sequence, charge balance, or introducing additional stabilizing interactions, so as to broaden the applicability of this platform in more physiologically relevant settings.

In conclusion, the proposed R10/DNA coacervate represents a promising biomimetic delivery platform that combines excellent biocompatibility with innovative biomacromolecular transport capabilities. Their transmembrane uptake mechanisms and controllable release properties offer valuable insights into cellular transport processes and have the potential to guide the design of next‐generation intelligent delivery systems. Further research focusing on carrier optimization, a deeper mechanistic understanding, and the establishment of robust preclinical evaluation models will be essential to advance their translational applications in biomedicine.

## 4 Methods

### 4.1 Materials

K10, R10, RA1, RA2, and FITC‐R10 peptides were purchased from GUOPING Pharmaceutical (Anhui, China). Low‐molecular‐weight DNA from salmon sperm and saporin peptides were purchased from Sigma‐Aldrich. Trypsin‐EDTA(1X) (0.25%) was purchased from Gibco. Opti‐MEM (Reduced‐Serum Medium), Dil, and LysoTracker Red DND‐99 were purchased from Thermo Fisher Scientific. R‐Phycoerythrin, MβCD, Chlorpromazine, and Cy3 were purchased from MedChemExpress. DNase I was obtained from Beyotime (Shanghai, China). FITC‐BSA was purchased from Life Sciences. EGFP was purchased from Maokang Bio (Shanghai, China). Calcein‐AM/PI Live/Dead Assay Kit was purchased from BestBio (Shanghai, China). PBS and Senescence‐Associated β‐Gal Stain Kit were purchased from SolarBio. Dulbecco's modified Eagle's medium (DMEM), Ham's F‐12K Nutrient Mixture (F‐12K), RPMI 1640, Minimum Essential Medium (MEM), and Cell Counting Kit‐8 were obtained from Fuheng Biotechnology (Shanghai, China). Cells were cultured in complete medium (with 10% FBS and 1% penicillin‐streptomycin).

### 4.2 Coacervation of Peptide and DNA and Recruitment of Cargo

Peptide, DNA, and cargo solutions were added directly into a centrifuge tube and mixed thoroughly, regardless of the order of addition. The peptide working solution was prepared in PBS at a concentration of 2 mg/mL, and the DNA solution was prepared in PBS at a concentration of 10 mg/mL. The mass ratio of peptide to DNA was adjusted from 1:5 to 5:1 depending on experimental requirements. FITC‐BSA, R‐PE, and EGFP were added at a fixed coacervate‐to‐cargo mass ratio of 20:1.

### 4.3 Characterization of Coacervates

For the measurement of relative turbidity of coacervates, 2 mg/mL peptide and 2 mg/mL DNA solutions were gently mixed at different volume ratios to obtain a 100‐µL mixture, and the absorbance at 600 nm was measured with a microplate reader to assess relative turbidity.

### 4.4 FRAP Data Processing and Quantitative Analysis

FRAP data were analyzed using ZEISS ZEN software, following standard FRAP analysis workflows. For each experiment, three regions were defined: (i) a region of interest (ROI) corresponding to the photobleached area, (ii) an unbleached reference region (positive control) to correct for acquisition‐related photobleaching, and (iii) a background region to account for camera and environmental noise (negative control). The bleaching depth was controlled to approximately 40% of the initial fluorescence intensity. Mean fluorescence intensities were extracted from each region at all time points, and the resulting recovery curves were subsequently processed and fitted.

### 4.5 Molecular Dynamics Simulations

All‐atom molecular dynamics simulations were performed using the AMBER14SB_OL15 force field together with the TIP4P‐FB water model. Each system was solvated in a cubic periodic box of 10 × 10 × 10 nm^3^, and neutralized with sodium and chloride ions parametrized consistently with the water model. After energy minimization and equilibration, production simulations were carried out for 2.5 µs with a 2 fs integration time step.

Temperature was maintained at 298.15 K using the V‐rescale thermostat, and pressure was controlled at 1 bar using the Parrinello–Rahman barostat. Long‐range electrostatic interactions were treated with the particle–mesh Ewald (PME) method, and short‐range nonbonded interactions were computed using the Verlet cutoff scheme. All bonds involving hydrogen atoms were constrained with LINCS. Trajectory coordinates and energies were saved every 10 ps.

For contact analysis, contacts between the peptide and DNA were defined using a 0.45 nm heavy‐atom distance cutoff. Contact numbers were computed for each frame along the trajectory, and normalized by the peptide length to yield the average number of contacts per residue per frame.

### 4.6 Cell Culture

Hek293T, OC‐1, and NIH‐3T3 cells were grown in complete DMEM at 37°C with 5% CO_2_. HeLa, A549, and HepG2 cell lines were grown separately in complete RPMI 1640, F‐12K complete medium, and MEM complete medium under identical conditions with 10% FBS and antibiotics.

### 4.7 Delivery of Proteins and Efficiency

For the delivery of coacervates and evaluation of delivery efficiency, A density of 2 × 10^4^ HeLa cells/well was seeded in 6‐well plates and cultured for 24 h in complete RPMI 1640. After aspirating the medium, cells were rinsed twice with PBS to eliminate residual serum components. Subsequently, cells were exposed to protein‐loaded coacervates (0.4 mg/mL) in 1 mL of Opti‐MEM for 4 h, washed once with 1 M NaCl solution, and subsequently rinsed twice with PBS (pH 7.4). Following trypsinization and two PBS washes, cell samples were measured on an Attune NxT flow cytometer configured for 488 nm excitation and 530/30 nm emission detection. Serum‐free Opti‐MEM was used for coacervate incubation. All cellular uptake experiments were conducted in serum‐free Opti‐MEM after removal of complete medium and washing to minimize residual serum components.

### 4.8 Colocalization of Lysosome and Coacervates

We seeded HeLa cells in 35‐mm confocal dishes at 1 × 10^4^ cells/dish in 1.5 mL of complete RPMI 1640 medium and allowed them to adhere for 24 h prior to experimentation. After incubation with Cy3‐labled coacervates (0.4 mg/mL) in 1 mL of Opti‐MEM (Reduced‐Serum Medium) for 4 h at 37°C, cells were washed once with 1 M NaCl solution and subsequently rinsed twice with PBS (pH 7.4). According to the manufacturer's instructions, the cells were stained with 100 nM LysoTracker in 1 mL of Opti‐MEM for 30 min at 37°C under 5% CO_2_, protected from light. Subsequently, the staining solution was replaced with 1 mL of fresh Opti‐MEM, and live‐cell imaging was immediately performed using a confocal laser scanning microscope (Leica SP8 LSCM) fitted with a 63× oil‐immersion objective. Fluorescence signals were captured with excitation/emission settings of 504/511‐nm using LysoTracker.

### 4.9 Internalization Mechanism

We seeded HeLa cells in 35‐mm dishes at the aforementioned density and let them attach for 24 h in 1.5 mL of complete medium. After aspirating the medium, cells were pretreated with 30 µM CPM or 4.5 mM MβCD for 4 h [16]. Subsequently, the cells were incubated with 1 mL of FITC‐BSA‐loaded R10/DNA coacervates in Opti‐MEM (0.4 mg/mL) for 4 h. The residual coacervates were removed by washing once with 1 M NaCl solution and twice with PBS (pH 7.4). Using 488 nm excitation and 530/30 nm emission detection on a flow cytometer, we quantified the fluorescence intensity. Control groups included totally untreated (Negative control) cells and cells treated with 0.4 mg/mL FITC‐BSA‐loaded coacervates without pretreating with inhibitors.

### 4.10 Degradation of Coacervates In Vitro

As recommended by the manufacturer, each reaction mixture was prepared by sequentially adding pre‐formed R10/DNA coacervates (5 µg, 4 mg/mL), 5 µL of 10× DNase I Reaction Buffer, and 2 µL of DNase I (1 U/µL) into 1.5 mL microcentrifuge tubes. A final volume of 50 µL was achieved by adding nuclease‐free water, and the mixtures were incubated at 37°C in a metal heating block for 0, 30, 60, or 120 min to assess the degradation of coacervates. The droplet integrity and morphological changes were monitored using an inverted phase‐contrast microscope. Control reactions lacking DNase I were performed by replacing the enzyme with an equal volume (2 µL) of nuclease‐free water.

### 4.11 Cytotoxicity Study

Cytotoxic effects of the coacervates without cargo were assessed by the CCK‐8 assay. DMEM (100 µL; with 10% FBS and antibiotics) containing 5 × 10^4^ OC‐1 cells/mL was dispensed into a 96‐well plate and incubated in the CO_2_ incubator (5% CO_2_, 37°C) for 24 h. After washing with PBS, DMEM was replaced with a coacervate suspension in serum‐free Opti‐MEM at various concentrations (0–0.7 mg/mL and incubated for 24–72 h. Cell viability was then assessed using the CCK‐8 kit, following the supplier's instructions. After 2 h of incubation, absorbance at 450 nm was recorded using a microplate reader. The calcein‐AM/PI double‐staining assay was also employed to assess cell viability. After 6 h incubation with 0.4 mg/mL coacervates in Opti‐MEM, cells were sequentially treated with Calcein‐AM and PI and observed using an inverted fluorescence microscope (Mshot).

### 4.12 Statistical Analysis

All data are presented as mean ± standard deviation (SD). The sample size (n) for each statistical analysis corresponds to the number of biologically independent replicates is specified in the corresponding figure legend. Statistical comparisons between two groups were performed using a two‑sided Student's *t*‑test. For comparisons involving more than two groups, one‑way analysis of variance (ANOVA) was used, followed by Tukey's posthoc test for multiple comparisons. Data were analyzed by two‑way ANOVA with two factors as independent variables, followed by Tukey's post‑hoc test for multiple comparisons when appropriate. Statistical significance was set at α = 0.05 (two‑sided). In all figures, significance levels are defined as: ns, not significant, ^*^ *p* < 0.05, ^**^ *p* < 0.01, ^***^ *p* < 0.001, ^****^ *p* < 0.0001. All statistical analyses were performed using Origin or GraphPad Prism 10. All microscopy images are representative of at least three independent experiments, all of which gave similar results.

## Conflicts of Interest

The authors declare no conflicts of interest.

## Supporting information




**Supporting File 1**: advs75691‐sup‐0001‐SuppMat.docx.

**Supporting File 2**: advs75691‐sup‐0002‐DataFile.xlsx.

## Data Availability

The data supporting the findings of this study are provided in the supplementary material accompanying this article.
