# Supplementary material for: Oligopeptides/DNA Coacervate Droplets as Macromolecular Delivery Microcarriers
Source: Adv Sci (Weinh). 2026 May 13;13(44):e75691. doi: 10.1002/advs.75691 (PMC13335948; doi:10.1002/advs.75691)
Supplement: Supplementary file 1 [file ADVS-13-e75691-s002.docx]

**Oligopeptides/DNA Coacervate Droplets as Macromolecular Delivery Microcarriers**

Linyi Zhang^1,#^, Chong Wang^2,#^, Mengqi Han^1^, Yi Yang^3,*^, Teng Ma^4,*^, Luoran Shang^1,*^

1 Shanghai Xuhui Central Hospital, Zhongshan-Xuhui Hospital, Shanghai Key Laboratory of Medical Epigenetics, International Co-laboratory of Medical Epigenetics and Metabolism (Ministry of Science and Technology, Institutes of Biomedical Sciences), Fudan University, Shanghai, China.

2 Department of Rheumatology and Immunology, Nanjing Drum Tower Hospital, School of Biological Science and Medical Engineering, Southeast University, Nanjing, China.

3 Department of Cardiovascular Surgery, Ruijin Hospital, Shanghai Jiao Tong University School of Medicine, Shanghai, China.

4 Department of Thoracic Surgery, Zhongshan Hospital, Fudan University, Shanghai, China.

Email: ma.teng@zs-hospital.sh.cn; yy12181@rjh.com.cn; luoranshang@fudan.edu.cn

#These authors contributed equally to this work

**Supplementary Fig. 1** Representative confocal microscopy images of R10/DNA coacervate droplets incubated in serum-free Opti-MEM with or without PEG-8000 at 37°C for the indicated times.

**Supplementary Fig. 2** Quantitative analysis of the average droplet area as a function of incubation time of R10/DNA coacervate. The experimental conditions are serum-free Opti-MEM without PEG-8000 and Opti-MEM containing 5% (w/v) PEG-8000. Data are presented as mean ± s.d. (n=177-1529). F-test was used for statistical analysis. ***P < 0.001.

**Supplementary Fig. 3** Confocal fluorescent images of Hela cells following 4 h of treatment with FITC-BSA-loaded R10/DNA coacervate droplets. Lysosomes were stained with LysoTracker.

**Supplementary Fig. 4** Confocal images of FITC-BSA loaded R10/DNA coacervates co-cultured with Lysotracker Red DND-99, demonstrating the enrichment of the dye in coacervate droplets.

**Supplementary Fig. 5** pH-dependent turbidity of R10/DNA coacervates. Relative turbidity of pre-formed R10/DNA coacervates immediately after exposure to buffers with pH values ranging from 4 to 9. Data are presented as mean ± s.d. (n = 3).

**Supplementary Fig. 6** Agarose gel electrophoresis showing degradation of salmon sperm DNA after incubation with nuclease (DNase I).

**Supplementary Fig. 7** **a**. Boxplots show droplets size distribution with or without DNase I (n=122). The box spans the interquartile range from the first to the third quartile, with the median indicated by the central line. Whiskers extend from the minimum to the maximum values. Two‑way ANOVA was used for statistical analysis. ****P < 0.0001. **b.** Number of droplets quantified from optical microscopy images with or without DNase I. Data are presented as mean ± s.d. (n = 3).

**Supplementary Fig. 8** Optical microscopy images of R10/DNA coacervate droplets formed at varying R10/DNA mass ratios after 2 hours of treatment with DNase I.

**Supplementary Fig. 9** Time-course confocal images of HeLa cells under fluorescence and bright-field views after exposure to free FITC-R10solution for up to 28 hours, demonstrating minimal fluorescence uptake.

**Supplementary Fig. 10** Confocal fluorescent microscopy images of Hela cells after co-cultured with dual-labeled coacervate droplets (FITC-R10/DNA coacervate droplets loaded with Cy3) at different time points.

**Supplementary Fig. 11** Confocal images of HeLa treated with free Cy3 solution for up to 4 hours, demonstrating minimal fluorescence uptake.
